# Gender-Specific Differences in Serum Sphingomyelin Species in Patients with Hepatitis C Virus Infection—Sphingomyelin Species Are Related to the Model of End-Stage Liver Disease (MELD) Score in Male Patients

**DOI:** 10.3390/ijms24098402

**Published:** 2023-05-07

**Authors:** Georg Peschel, Kilian Weigand, Jonathan Grimm, Martina Müller, Sabrina Krautbauer, Marcus Höring, Gerhard Liebisch, Christa Buechler

**Affiliations:** 1 Department of Internal Medicine I, University Hospital Regensburg, 93053 Regensburg, Germany; georg.peschel@klinikum-ffb.de (G.P.); kilian.weigand@gk.de (K.W.); jonathan.grimm@stud.uni-regensburg.de (J.G.); martina.mueller-schilling@klinik.uni-regensburg.de (M.M.); 2 Department of Internal Medicine, Klinikum Fürstenfeldbruck, 82256 Fürstenfeldbruck, Germany; 3 Department of Gastroenterology, Gemeinschaftsklinikum Mittelrhein, 56073 Koblenz, Germany; 4 Institute of Clinical Chemistry and Laboratory Medicine, University Hospital Regensburg, 93053 Regensburg, Germany; sabrina.krautbauer@klinik.uni-regensburg.de (S.K.); marcus.hoering@klinik.uni-regensburg.de (M.H.); gerhard.liebisch@klinik.uni-regensbug.de (G.L.)

**Keywords:** HCV genotype, direct-acting antivirals, liver cirrhosis, type 2 diabetes, MELD score, gender

## Abstract

Hepatitis C virus (HCV) replication depends on cellular sphingomyelin (SM), but serum SM composition in chronic HCV infection has been hardly analyzed. In this work, 18 SM species could be quantified in the serum of 178 patients with chronic HCV infection before therapy with direct-acting antivirals (DAAs) and 12 weeks later, when therapy was completed. Six SM species were higher in the serum of females than males before therapy and nine at the end of therapy; thus, sex-specific analysis was performed. Type 2 diabetes was associated with lower serum levels of SM 36:2;O2 and 38:2;O2 in men. Serum SM species did not correlate with the viral load in both sexes. Of note, three SM species were lower in males infected with HCV genotype 3 in comparison to genotype 1 infection. These SM species normalized after viral cure. SM 38:1;O2, 40:1;O2, 41:1;O2, and 42:1;O2 (and, thus, total SM levels) were higher in the serum of both sexes at the end of therapy. In males, SM 39:1;O2 was induced in addition, and higher levels of all of these SM species were already detected at 4 weeks after therapy has been started. Serum lipids are related to liver disease severity, and in females 15 serum SM species were low in patients with liver cirrhosis before initiation of and after treatment with DAAs. The serum SM species did not correlate with the model of end-stage liver disease (MELD) score in the cirrhosis and the non-cirrhosis subgroups in females. In HCV-infected male patients, nine SM species were lower in the serum of patients with cirrhosis before DAA treatment and eleven at the end of the study. Most of the SM species showed strong negative correlations with the MELD score in the male cirrhosis patients before DAA treatment and at the end of therapy. Associations of SM species with the MELD score were not detected in the non-cirrhosis male subgroup. In summary, the current analysis identified sex-specific differences in the serum levels of SM species in HCV infection, in liver cirrhosis, and during DAA therapy. Correlations of SM species with the MELD score in male but not in female patients indicate a much closer association between SM metabolism and liver function in male patients.

## 1. Introduction

Chronic hepatitis C virus (HCV) infection is a major cause of serious liver diseases and bears an increased risk for acquiring extrahepatic manifestations such as cardiovascular diseases or diabetes [1,2].

HCV relies on lipids for its reproduction and infection. Depletion of viral cholesterol or treatment of viral particles with sphingomyelinase abolished HCV infection [3]. Blockage of serine palmitoyltransferase, which catalyzes the first step in sphingolipid biosynthesis, hindered HCV replication in hepatocytes and experimental models [4,5,6]. A serine palmitoyltransferase inhibitor effectively blocked the replication of HCV genotypes 1a, 1b, 2a, 3a, and 4a in mice with humanized livers [5]. 

Sphingomyelin synthase (SMS) catalyzes the conversion of phosphatidylcholine and ceramide to sphingomyelin and diacylglycerol, and HCV infection is related to the higher expression of SMS1 and SMS2 in hepatocytes of humanized chimeric mice [7]. 

HCV genotype 1a and 2a infection of mice increased sphingomyelin levels in the hepatocytes [8]. Sphingomyelin (SM) 42:2;O2 was elevated in genotype 1a and SM 34:1;O2 and 42:2;O2 were higher in genotype 2a infection in comparison to uninfected mice [8]. The acyl-chain lengths of SMs affect cell membrane structure and function [9] as well as the endocytic sorting of lipids [10] suggesting that the HCV genotype-2a-specific induction of SM 34:1;O2 may contribute to HCV genotype differences on disease pathology [11].

SMs are highly abundant in serum and represent 50% of the lipids carried by low-density lipoprotein (LDL) and 42% of high-density lipoprotein (HDL)-contained lipids. SM 34:1;O2 and 42:2;O2 are the dominant circulating species [12,13]. In the healthy population SM 36:0;O2 and SM 36:2;O2 were higher in female compared to male plasma [12].

The SM content in plasma regulates cholesteryl ester (CE) composition. A high plasma SM/phosphatidylcholine ratio was associated with lower percentages of cholesteryl ester (CE) species 20:4, 22:5, and 22:6, which are all formed by lecithin cholesterol acyltransferase (LCAT), and higher percentages of CE 18:1. The higher abundance of CE species with shorter acyl chains and less double bonds may contribute to the pro-atherogenic effects of high plasma SM levels [14].

Circulating SM species are also correlated with markers of liver function. SM 38:1;O2, 40:1;O2, and 42:1;O2 were positively related with aminotransferase levels indicative of an association with liver health [15]. However, in HCV patients with severe liver fibrosis serum SM species were not associated with aminotransferase levels [16].

There is convincing evidence that HCV infection is associated with diminished serum LDL levels [17,18]. Rapid normalization of LDL shortly after the start of an anti-viral therapy strongly indicates viral infection as the cause of hypolipidemia [19,20,21].

Acid sphingomyelinase (ASM) catalyzes the hydrolysis of SM to ceramide, and ASM activity in serum was higher in patients with chronic HCV compared to healthy controls. Sphingosine species were found to be increased in the serum of HCV-infected patients, but their levels did not correlate with aminotransferase activities, bilirubin levels, or HCV viral load [22].

Direct-acting antiviral (DAA) therapy is very effective and cures almost all patients from HCV [23]. Upon successful treatment of HCV systemic inflammation improves and levels of circulating inflammatory cytokines and chemokines decline [24,25,26]. Eradication of the virus is associated with the rise of serum LDL levels [19,21,25,27]. The effect of viral cure on serum SM species composition has, however, not been studied in detail as far as we know.

Lipid metabolism is greatly disturbed in patients with advanced liver fibrosis. LDL and total serum cholesterol levels are low in patients with severe liver diseases [28]. Liver-fibrosis-related variations of circulating SM species have been hardly analyzed so far. A comparison of HCV-infected patients with fibrosis grade F1–F2 to those with F5–F6 scores, according to an Ishak score, did not identify a significant change in serum SM species [16]. In hepatitis-B-infected patients, serum SM 42:1;O2 negatively correlated with the model for end-stage liver disease (MELD) score [29]. In patients with predominantly alcoholic liver cirrhosis seven of the nineteen measured SM species were negatively related to the MELD score [30]. While these studies suggest that SM species decline in patients with advanced liver fibrosis it was also shown that SM 36:0,O2 was higher in the serum of patients with HCV and advanced fibrosis stages in comparison to patients without fibrosis [31].

Taken together, there is sufficient evidence that HCV infection as wells as advanced liver injury disturb SM homeostasis. The aim of this study was to identify associations of different serum SM species with markers of liver disease severity. HCV-infection-related lipid abnormalities generally resolve after elimination of the virus [32] and comparison of SM levels before and after virus eradication enables the identification of the SM species changed by HCV infection. Findings from this study will contribute to better understanding SM metabolism in chronic liver diseases. 

## 2. Results

### 2.1. Associations of Sphingomyelin Species with Sex, Age, Body Mass Index, Liver Steatosis, and Type 2 Diabetes in HCV Patients

**Figure 1:**
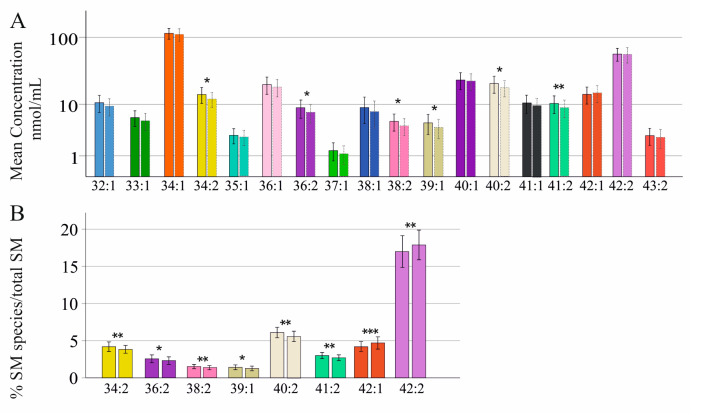
Sphingomyelin (SM) species in relation to gender in patients with chronic HCV infection: (**A**) SM species in 74 female (left bar) and 104 male (right bar) patients. Mean concentration ± standard deviation is shown; (**B**) SM species in % of total SM levels, which differed between sexes in female (left bar) and male (right bar) patients. * *p* < 0.05, ** *p* < 0.01, *** *p* < 0.001.

Sphingomyelins (SMs), which are also referred to as sphingophospholipids, are highly abundant serum lipids and consist of a sphingosine base (typically 18 carbon chains and one double bond (C18:1), C16, and C20 sphingoid bases are also occurring in human serum) attached to a phosphorylcholine and an N-linked fatty acid with different numbers of carbons. Hence, SM 34:1;O2 may represent a sphingoid base with 18 carbon atoms, one double bond, and two hydroxyl groups and a N-acyl chain with 16 C-atoms (Supplementary Figure S1) [33,34]. Here, 18 SM species, 32:1;O2, 33:1;O2, 34:1;O2, 34:2;O2, 35:1;O2, 36:1;O2, 36:2;O2, 37:1;O2, 38:1;O2, 38:2;O2, 39:1;O2, 40:1;O2, 40:2;O2, 41:1;O2, 41:2;O2, 42:1;O2, 42:2;O2, and 43:2;O2, were measured in the sera of 178 patients with chronic HCV infection, and this cohort was already described [35]. Levels of SM 34:2;O2, 36:2;O2, 38:2;O2, 39:1;O2, 40:2;O2, and 41:2;O2 were lower in the serum of the 104 male in comparison to the 74 female patients (Figure 1A). Percent SMs 34:2;O2, 36:2;O2, 38:2;O2, 39:1;O2, 40:2;O2, and 41:2;O2 were higher in female patients, and the relative content of SM 42:1;O2 and 42:2;O2 were lower in females in comparison to male subjects (Figure 1B). Total serum SM levels did not vary between sexes (*p* = 0.072). SM species did not differ between normal-weight and overweight patients and did not correlate with BMI or age in both sexes (*p* > 0.05). 

Seventy-four patients had liver steatosis, which was not associated with altered concentrations of serum SM species in the whole cohort and when both sexes were analyzed separately (*p* > 0.05 for all). SM levels did not differ between diabetic and non-diabetic patients in the whole cohort and in female patients (Supplementary Table S1). In males, diabetic patients had lower serum levels of SM 36:2;O2 and SM 38:2;O2 (Supplementary Table S1).

### 2.2. Sphingomyelin Species in Female Patients with and without Liver Cirrhosis Diagnosed by Ultrasound or the Fibrosis-4 Score

**Figure 2:**
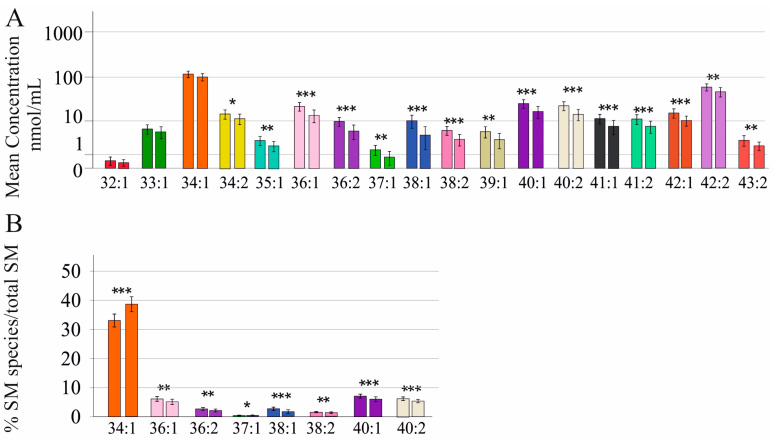
Sphingomyelin (SM) species in relation to liver cirrhosis analyzed by ultrasound in female patients with chronic HCV: (**A**) SM species in the serum of 58 females without (left bar) and 16 females with ultrasound-diagnosed liver cirrhosis (right bar). Mean concentration ± standard deviation is shown; (**B**) SM species different in cirrhosis are shown as a % of total SM levels (left bar: female patients without cirrhosis; right bar: female patients with cirrhosis). * *p* < 0.05, ** *p* < 0.01, *** *p* < 0.001.

SM species levels in serum differed between sexes (Figure 1A). Therefore, the calculation of cirrhosis-associated SM alterations was done separately for female and male patients. In females all but SM 32:1;O2, 33:1;O2, and 34:1;O2 were reduced in cirrhosis diagnosed by ultrasound examination (Figure 2A). Relative to total SM levels, SM 36:1;O2, 36:2;O2, 37:1;O2, 38:1;O2, 38:2;O2, 40:1;O2, and 40:2;O2 were lower and % SM 34:1;O2 was higher in cirrhosis (Figure 2B). 

The fibrosis-4 (FIB-4) score is appropriate to distinguish between HCV patients without liver fibrosis and patients with advanced fibrosis; the diagnosis of intermediate fibrosis stages using the FIB-4 score is less accurate [36]. None of the SM species differed between patients with low and intermediate scores. All but SM 32:1;O2, 33:1;O2, 34:1;O2, and 43:2;O2 were lower in patients with fibrosis in comparison to patients without fibrosis (Supplementary Table S2). All but SM 32:1;O2 and 33:1;O2 were reduced in the serum of female patients with fibrosis in comparison to female patients with intermediate fibrosis scores (Supplementary Table S2).

The levels of serum SM species did not correlate with alanine aminotransferase (ALT), aspartate aminotransferase (AST), bilirubin, C-reactive protein (CRP), leukocytes, platelets, or creatinine in female patients with or without liver cirrhosis (Supplementary Tables S3 and S4). In non-cirrhosis patients a negative correlation of SM 34:2;O2 with international normalized ratio (INR) and the MELD score was detected (Supplementary Table S3). In cirrhosis SM 36:2;O2 (r = 0.815, *p* < 0.01) and 38:2;O2 (r = 0.768, *p* < 0.05) positively correlated with albumin levels (Supplementary Table S4). It is worth noting that these SM species correlated with HDL levels (Supplementary Table S5). When adjusted for HDL levels, the correlations of SM 36:2;O2 and 38:2;O2 with albumin were not significant (*p* > 0.05).

### 2.3. Sphingomyelin Species in Male Patients with and without Liver Cirrhosis Diagnosed by Ultrasound or the Fibrosis-4 Score

**Figure 3:**
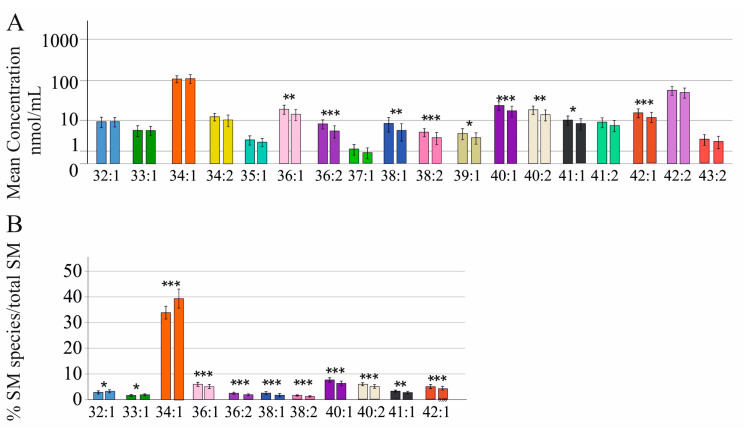
Sphingomyelin (SM) species in relation to liver cirrhosis diagnosed using ultrasound in male patients with chronic HCV infection: (**A**) SM species in serum of 80 males without (left bar) and 24 males with ultrasound-diagnosed liver cirrhosis (right bar). Mean concentration ± standard deviation is shown; (**B**) SM species differing in cirrhosis are shown as a % of total SM levels (left bar: males without liver cirrhosis; right bar: males with liver cirrhosis). * *p* < 0.05, ** *p* < 0.01, *** *p* < 0.001.

In males SM 32:1;O2, 33:1;O2, 34:1;O2, 34:2;O2, 35:1;O2, 37:1;O2, 41:2;O2, 42:2;O2, and 43:2;O2 levels were not reduced in cirrhosis, which was diagnosed using ultrasound, whereas the further nine analyzed SM species declined (Figure 3A). Relative to total SM levels SM 32:1;O2, 33:1;O2, and 34:1;O2 were higher, and SM 36:1;O2, 36:2;O2, 38:1;O2, 38:2;O2, 40:1;O2, 40:2;O2, 41:1;O2, and 42:1;O2 were lower in cirrhosis (Figure 3B). 

SMs 32:1;O2, 33:1;O2, 34:1;O2, 34:2;O2, 35:1;O2, 37:1;O2, 39:1;O2, 41:1;O2, 41:2;O2, 42:2;O2, and 43:2;O2 were not changed in fibrosis defined by the FIB-4 score. SMs 36:1;O2, 36:2;O2, 38:1;O2, 38:2;O2, 40:1;O2, 40:2;O2, and 42:1;O2 were reduced in patients with a high FIB-4 score compared to those with a low score. SM 36:2;O2 and 38:1;O2 differed between patients with an intermediate and a high score. Patients with low and intermediate FIB-4 scores had comparable SM species levels (Supplementary Table S6).

In male patients with liver cirrhosis, serum SMs 36:1;O2, 36:2;O2, 37:1;O2, 38:1;O2, 38:2;O2, 39:1;O2, 40:1;O2, 40:2;O2, 41:1;O2, 42:1;O2, and 43:2;O2 negatively correlated with INR and the MELD score (Table 1). SM 34:2;O2 was negatively related to the MELD score, whereas the association with INR was not significant (Table 1). SM38:1;O2 was negatively associated with bilirubin. None of the SM species were associated with ALT, AST, albumin, CRP, leukocytes, platelet count, or creatinine in male patients with liver cirrhosis (Supplementary Table S7). 

SM is part of HDL and LDL particles, and strong positive correlations of all but SM 34:1;O2 with LDL were identified in males (Supplementary Table S5). LDL is reduced in cirrhosis and negatively correlated with the MELD score (r = −0.612, *p* = 0.040); after correction for LDL as a confounding factor, the correlations of SM species with INR and the MELD score were no longer significant (*p* > 0.05 for all).

In non-cirrhosis patients, none of the SM species correlated with the MELD score, ALT, AST, bilirubin, albumin, INR, CRP, leukocytes, platelets, or creatinine (Supplementary Table S8).

It is important to note that total serum SM levels were similar between sexes in the group of patients with liver cirrhosis (*p* = 0.440) and in patients without liver cirrhosis (*p* = 0.16).

### 2.4. Sphingomyelin Species Levels in Relation to HCV Viral Load and Genotype

**Figure 4:**
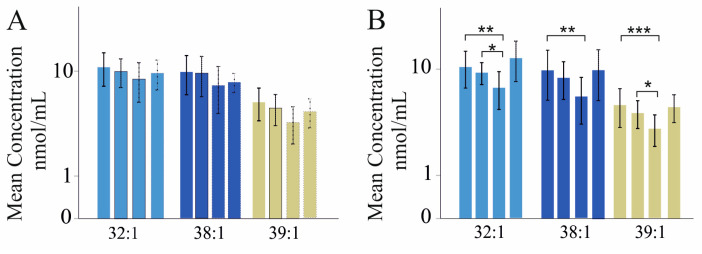
Sphingomyelin (SM) species in relation to genotype in patients with chronic HCV infection. Bars, from left to the right, correspond to genotype 1a, 1b, 3a, and rare genotypes. (**A**) SM species 32:1;O2, 38:1;O2, and 39:1;O2 in serum of females infected with genotype 1a, 1b, 3a, or rare genotypes; (**B**) SM species 32:1;O2, 38:1;O2, and 39:1;O2 in the serum of males infected with genotype 1a, 1b, 3a, or rare genotypes. Mean concentration ± standard deviation is shown. * *p* < 0.05, ** *p* < 0.01, *** *p* < 0.001.

None of the SM species was associated with HCV viral load in patients with and without cirrhosis of both sexes (*p* > 0.05 for all). Because of the decrease in several serum SM species in patients with liver cirrhosis, associations with viral genotypes were calculated in HCV patients without liver cirrhosis. In female patients, 19 patients had genotype 1a, 23 patients had genotype 1b, 7 patients had genotype 3a, and 9 patients had rare genotypes such as 2 or 2a. In male patients, 24 had genotype 1a, 30 genotype 1b, 22 patients had genotype 3a, and 4 patients had rare genotypes. None of the SM species differed by genotype in females (Figure 4A). Genotype-3a-infected males had lower SM 32:1;O2, 38:1;O2, and 39:1;O2 levels than 1a-infected male patients and lower SM 32:1;O2 and 39:1;O2 concentrations than 1b-infected men (Figure 4B). 

### 2.5. Effect of DAA Therapy on Serum Sphingomyelin Levels

**Figure 5:**
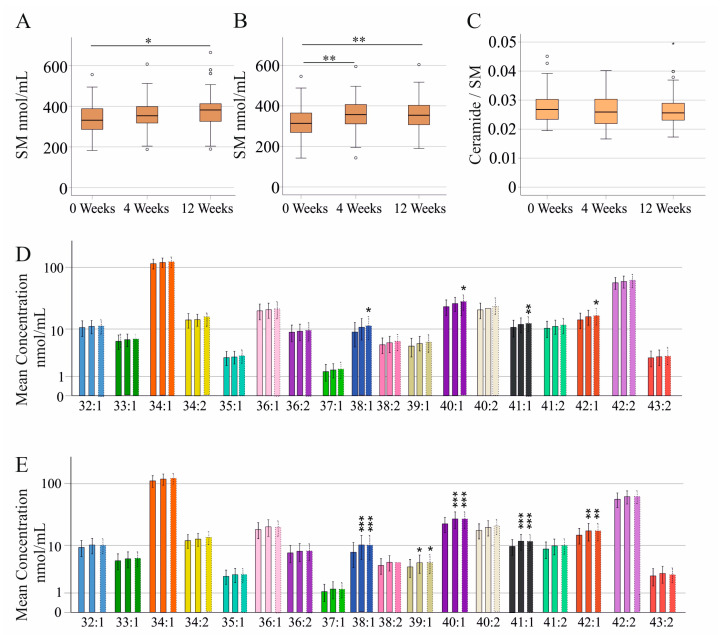
Total sphingomyelin (SM) levels and SM species during the study: (**A**) Total SM levels in females during the study; (**B**) Total SM levels in males during the study; (**C**) Ceramide/SM ratio during the study; (**D**) SM species in female patients before therapy (0 weeks; 178 patients, left bar), at 4 weeks (178 patients, middle bar), and 12 weeks (176 patients, right bar) after start of treatment; (**E**) SM species in male patients before therapy (0 weeks; 178 patients, left bar), at 4 weeks (178 patients, middle bar), and 12 weeks (176 patients, right bar) after start of treatment. * *p* < 0.05, ** *p* < 0.01, *** *p* < 0.001 for the comparison to 0 weeks.

DAA therapy is very effective, and the viral load was significantly reduced at 4 weeks of therapy in both sexes (Supplementary Figure S2). Total SM levels increased during DAA therapy in females and males (Figure 5A,B). In males, a significant increase was already noticed at 4 weeks after therapy has been started (Figure 5B). In females, the difference between pretreatment levels of total SM and levels at the end of therapy was significant. Notably, the ceramide/SM ratio did not change during therapy in the whole cohort (Figure 5C) and when both sexes were analyzed separately (*p* > 0.05).

Serum cholesterol concentrations recovered similarly in males and females and at the end of therapy were significantly higher in both sexes (in females cholesterol levels were 111% of the levels before therapy, *p* = 0.038, and in males 115% of levels before therapy, *p* < 0.001). Moreover, viral titer was significantly decreased at 4 weeks after the start of therapy in both sexes (*p* < 0.001 for males and females; Supplementary Figure S2). Thus, faster normalization of serum SM levels in males is not associated with more rapid elimination of the virus or earlier recovery of serum cholesterol.

Regarding single SM species, SMs 38:1;O2, 40:1;O2, 41:1;O2, and 42:1;O2 were higher at the end of therapy in female patients (Figure 5D). In male patients SMs 38:1;O2, 39:1;O2, 40:1;O2, 41:1;O2, and 42:1;O2 were already significantly higher at 4 weeks after therapy started and remained increased until therapy ended (Figure 5E). 

At the end of therapy the levels of SM 32:1;O2, 33:1;O2, 34:2;O2, 36:2;O2, 37:1;O2, 38:2;O2, 39:1;O2, 40:2;O2, and 41:2;O2 were lower in the serum of male than female patients (*p* < 0.05). Percent SMs 34:2;O2, 36:2;O2, 37:1;O2, 38:2;O2, 39:1;O2, 40:2;O2, and 41:2;O2 were higher in females and the relative content of SM 42:1;O2 and 42:2;O2 was reduced in comparison to males (*p* < 0.05). Total serum SM levels did not vary between sexes (*p* > 0.05). 

In the whole cohort SM 40:1;O2 (r = −0.247, *p* = 0.014) and 42:1;O2 (r = −0.287, *p* = 0.002) negatively correlated with age. These associations were not found in sex-specific analysis. SM species did not correlate with BMI in the whole cohort, in male and in female patients (*p >* 0.05).

### 2.6. Sphingomyelin Species Levels in Female Patients with and without Liver Cirrhosis Diagnosed by Ultrasound at Therapy End

**Figure 6:**
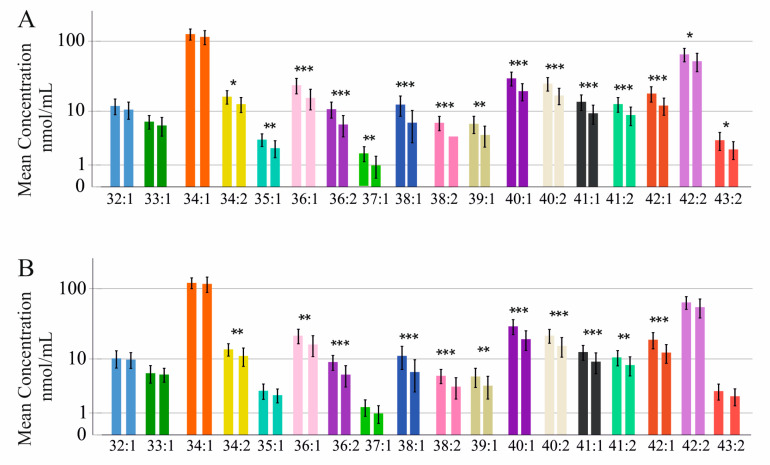
Sphingomyelin (SM) species in relation to liver cirrhosis diagnosed using ultrasound: (**A**) SM species in the serum of females without (left bar) and with ultrasound-diagnosed liver cirrhosis (right bar); (**B**) SM species in the serum of males without (left bar) and with ultrasound-diagnosed liver cirrhosis (right bar). * *p* < 0.05, ** *p* < 0.01, *** *p* < 0.001.

At the end of therapy in the serum of female patients all but SMs 32:1;O2, 33:1;O2, and 34:1;O2 were lower in cirrhosis, which was diagnosed via ultrasound examination (Table 2 and Figure 6). Relative to total SM levels, % SMs 36:1;O2, 36:2;O2, 37:1;O2, 38:1;O2, 38:2;O2, 40:1;O2, 40:2;O2, 41:1;O2, 41:2;O2, and 42:1;O2 were lower and % SM 34:1;O2 was higher in cirrhosis (Supplementary Table S9). 

Serum SM species levels did not correlate with the MELD score, ALT, AST, bilirubin, albumin, INR, CRP, leukocytes, platelet count, or creatinine in female patients without liver cirrhosis (*p* > 0.05). In cirrhosis, negative correlations of SM 36:2;O2 (r = −0.719, *p* = 0.024), SM 38:2;O2 (r = −0.724, *p* = 0.022), and SM 40:1;O2 (r = −0.768, *p* = 0.007) with bilirubin were observed. SM 37:1;O2 (r = 0.708, *p* = 0.031) was positively associated with albumin. 

### 2.7. Sphingomyelin Species Levels in Male Patients with and without Liver Cirrhosis Diagnosed by Ultrasound at End of Therapy

In male patients all but SMs 32:1;O2, 33:1;O2, 34:1;O2, 35:1;O2, 37:1;O2, 42:2;O2, and 43:2;O2 were reduced in cirrhosis diagnosed by ultrasound examination (Table 3 and Figure 6). Relative to total SM levels, SMs 32:1;O2, 33:1;O2, and 34:1;O2 were higher and % SMs 36:1;O2, 36:2;O2, 38:1;O2, 38:2;O2, 40:1;O2, 40:2;O2, 41:1;O2, and 42:1;O2 were lower in cirrhosis (Supplementary Table S10). 

Serum SM species levels did not correlate with the MELD score, ALT, AST, bilirubin, albumin, INR, CRP, leukocytes, platelets, or creatinine in male patients without liver cirrhosis (*p* > 0.05). 

In the 24 male cirrhosis patients negative correlations of SMs 34:2;O2 (r = −0.664, *p* = 0.008), 35:1;O2 (r = −0.675, *p* = 0.006), 36:1;O2 (r = −0.675, *p* = 0.006), 36:2;O2 (r = −0.727, *p* < 0.001), 37:1;O2 (r = −0.792, *p* < 0.001), 38:1;O2 (r = −0.748, *p* = 0.001), 38:2;O2 (r = −0.816, *p* < 0.001), 39:1;O2 (r = −0.791, *p* < 0.001), 40:1;O2 (r = −0.804, *p* < 0.001), 40:2;O2 (r = −0.808, *p* < 0.001), 41:1;O2 (r = −0.792, *p* < 0.001), 41:2;O2 (r = −0.783, *p* < 0.001), and 42:1;O2 (r = −0.726, *p* = 0.001) with the MELD score were identified.

SMs 33:1;O2 (r = −0.593, *p* = 0.047), 35:1;O2 (r = −0.742, *p* = 0.001), 36:1;O2 (r = −0.718, *p* = 0.002), 36:2;O2 (r = −0.637, *p* = 0.017), 37:1;O2 (r = −0.793, *p* < 0.001), 38:1;O2 (r = −0.762, *p* < 0.001), 38:2;O2 (r = −0.660, *p* = 0.009), 39:1;O2 (r = −0.839, *p* < 0.001), 40:1;O2 (r = −0.765, *p* < 0.001), 40:2;O2 (r = −0.737, *p* = 0.001), 41:1;O2 (r = −0.771, *p* < 0.001), 41:2;O2 (r = −0.751, *p* < 0.001), and 42:1;O2 (r = −0.633, *p* = 0.019) correlated with INR. 

SM 37:1;O2 (r = −0.600, *p* = 0.041), 38:2;O2 (r = −0.669, *p* = 0.007), and 40:2;O2 levels (r = −0.603, *p* = 0.038) were related to bilirubin. 

## 3. Discussion

Here we show that the serum SM composition of patients with chronic HCV infection is gender-specific and differs between sexes before and after DAA therapy. Serum SM species levels were related to the MELD score in male patients with liver cirrhosis but not in the respective female cohort. This suggests a close relation between SM metabolism and liver function in male patients. Thus, distinct SM species are potential new biomarkers for liver injury in male HCV patients before and after DAA therapy. 

Sex-specific differences in serum SM species levels have been described before. In healthy controls 12 different SM species were analyzed, and SM 36:0;O2 and SM 36:1;O2 were higher in females than males [12]. This study enrolled five males and five females and not all differences may have been detected because of the small study group. In the serum of healthy females seven of the twenty-two analyzed SM species were higher in comparison to men, and this study enrolled fifteen males and fifteen females [37]. The current analysis showed that levels of SM 34:2;O2, 36:2;O2, 38:2;O2, 39:1;O2, 40:2;O2, and 41:2;O2 were increased in the serum of female compared to male HCV patients before therapy. At 12 weeks, these SM species and SMs 32:1;O2, 33:1;O2, and 37:1;O2 were higher in the serum of female compared to male patients. With the exception of SM 36:1;O2 all of these SM species were found to be increased in the plasma of healthy females in comparison to males [38]. Because the highly abundant serum SM species SM 34:1;O2 and 42:2;O2 were similar between sexes, total SM levels did not significantly differ in our cohort. 

Sex-specific differences in less-abundant SM species indicate a role for sex hormones in the regulation of their circulating levels. Hormonal contraceptives were shown to cause higher levels of four SM species, but only two of them differed between sexes [38] suggesting that further pathways contribute to sex-specific differences in circulating SM species levels.

The SM species in serum did not change with higher body weight in the current cohort, and this has also been reported in a group of obese patients for total serum SM levels [39]. A rise of SM 36:1;O2 and 40:1;O2 was detected in the serum of obese adults in comparison to lean controls [15]. Serum SM 40:1;O2 was also positively related to liver steatosis [40], which is more common in obesity. Positive correlations of SM 34:3 with BMI were observed in healthy controls [41]. Further study is needed to clarify whether any SM species is consistently associated with higher BMI, and if these associations are lost in HCV infection.

Whether serum SM composition changes with age is a further unresolved issue. In the current cohort of HCV patients, SM species did not correlate with age before therapy. Weak negative associations of SM 40:1;O2 and SM 42:1;O2 with age were detected at the end of therapy. However, in healthy humans 13 of the 19 analyzed SM species were positively associated with age [41].

In male HCV patients with type 2 diabetes serum levels of SM 36:2;O2 and SM 38:2;O2 were modestly reduced. It has been also shown that SM 36:1;O2 and 36:2;O2 are higher in the serum of type 2 diabetic patients in comparison to matched controls [42]. A further study described lower total SM levels in patients with diabetes [39]. The Homeostatic Model Assessment for Insulin Resistance did not, however, correlate with any of the 19 SM species measured in the serum of healthy controls [41]. The investigations published so far obtained discordant results and, moreover, mostly did not perform sex-specific analysis. At the moment, it cannot be concluded which of the SM species are specifically changed in type 2 diabetes. 

Though HCV infection uses the host’s SM metabolism [6], none of the SM species correlated with viral titer. SMs 38:1;O2, 40:1;O2, 41:1;O2, and 42:1;O2 were higher at the end of therapy in both sexes and this indicates that HCV infection causes a decline in specific SM species in both sexes. The recovery of SM species during DAA treatment was faster in males than in females. Serum cholesterol normalization was, however, similar in both sexes and viral titer was significantly decreased at 4 weeks after therapy started in males and females. This shows that a faster increase of serum SM in male patients is not related to the recovery of cholesterol or elimination of HCV. Liver function does not improve during DAA therapy [25,43], and further research is needed to clarify the processes involved. Moreover, three different SM species were associated with viral genotypes in males and none in females. Faster normalization of serum SM levels in males and sex-differences in HCV-genotype-related effects show that HCV-related changes in SM metabolism differ between males and females. 

In females with liver cirrhosis (diagnosed by ultrasound), of the 18 measured SM species, all but SM 32:1;O2, 33:1;O2, and 34:1;O2 declined. These species were also low when cirrhosis was defined by the FIB-4 score. Nine SM species, which were found to be reduced in females with cirrhosis, declined in males with ultrasound-diagnosed liver cirrhosis and seven were reduced in male patients with FIB-4 score-defined fibrosis. These data illustrate that advanced liver fibrosis has a stronger effect on serum SM composition in females than males. The decline in several SM species in non-alcoholic fatty liver disease patients with advanced fibrosis has been reported, but sex-specific analysis was not performed in this study [44]. In both sexes, SM species with longer acyl-chains declined in cirrhosis, whereas SM species with short acyl-chains remained the same as in patients without liver cirrhosis. The acyl-chain lengths of SM species are supposed to change cell membrane composition and function [9]; unravelling the effect of a shift from SM species with longer acyl-chain lengths to shorter variants is a challenge for the future.

Positive associations of SM 38:1;O2, 40:1;O2, and 42:1;O2 with AST and ALT were identified in young obese adults [15]. In the serum of patients with non-alcoholic steatohepatitis SMs 36:0;O2 38:0;O2, and 41:2;O2 were higher compared to patients with non-alcoholic steatosis [45]. Though these data suggest that serum SM species are positively related to measures of liver disease severity, associations with ALT and AST existed neither in females nor males in our HCV patient cohort. 

In females with liver cirrhosis SM 36:2;O2 and 38:2;O2 positively correlated with albumin levels before DAA therapy. At the end of therapy negative correlations of SM 36:2;O2, SM 38:2;O2, and SM 40:1;O2 with bilirubin were observed. Serum levels of these SM species as well as bilirubin and albumin did not significantly change during DAA treatment in the HCV cohort studied herein [35]. Whether these correlations are of pathophysiological importance needs further investigations.

The situation is quite different in male patients. In cirrhosis, serum SM 34:2;O2, 36:1;O2, 36:2;O2, 37:1;O2, 38:1;O2, 38:2;O2, 39:1;O2, 40:1;O2, 40:2;O2, 41:1;O2, and 42:1;O2 negatively correlated with the MELD score before and after DAA therapy. Most of the SM species, namely serum SM 36:1;O2, 36:2;O2, 37:1;O2, 38:1;O2, 38:2;O2, 39:1;O2, 40:1;O2, 40:2;O2, 41:1;O2, and 42:1;O2, negatively correlated with INR. SM 38:1;O2, 40:1;O2, 41:1;O2, and 42:1;O2 increased during DAA treatment but still correlated with INR and the MELD score at the end of therapy. INR is a measure of blood clotting time and is related to hepatic synthesis and functional capacity and, thus, is increased in cirrhosis [46]. The strong associations of several SM species with INR in males indicate that liver function is quite important for the serum levels of these SM species in males.

SM content of LDL and HDL particles is similar [13], and it is well known that cirrhosis is associated with lower serum lipid levels [18,28,47]. In females with liver cirrhosis, the three SM species found to be related to albumin also correlated with HDL. When adjusted for HDL levels the associations between albumin and the SM species were no longer significant arguing against a direct role of these SM species for blood albumin concentrations.

In male patients with liver cirrhosis almost all SM species positively correlated with LDL. Associations of these SM species with INR and the MELD score were lost after correcting for LDL levels. Thus, the correlation of SM species with the MELD score is related to the disturbed LDL metabolism in male patients with liver cirrhosis. Importantly, LDL negatively correlated with the MELD score in males but not females.

However, it may be too simplistic considering disturbed SM metabolism as a sole consequence of cholesterol homeostasis. SM in serum affects cholesterol metabolism, and high SM was shown to inhibit the LCAT reaction [14]. Enrichment of HDL with SM reduced cholesterol esterification by competition with phosphatidylcholine, the acyl donor for cholesterol esterification. Thus, a high SM/phosphatidylcholine ratio was related to lower CEs 20:4, 22:5, and 22:6, which are the product of LCAT, and higher CE 18:1, which is derived from acyl-coenzyme A:cholesterol acyltransferase (ACAT) [34]. In the serum of the HCV patients studied herein, CEs 18:1, 20:4, 20:5, and 22:6 were increased after DAA therapy [48]. This shows that the rise of serum SM levels could not prevent the increase of CEs 20:4 and 22:6 suggesting a greater effect of HCV elimination than higher SM levels on CE metabolism. 

LDL itself has sphingomyelinase activity, an enzyme that hydrolysis sphingomyelinase to yield ceramide [49]. The studies described above show that SM content affects cholesterol and lipoprotein metabolism and vice versa. The role of different SM species herein has, however, not been studied in detail. It has been known for a long time that lipid metabolism differs between sexes [50], which has been rarely addressed in patients with HCV infection and in patients with liver cirrhosis making it difficult to compare previous findings and the current study.

This study has limitations. Healthy controls were not enrolled, and the serum of HCV patients was not collected in the fasted state. Thus, these findings may not apply to HCV cohorts where fasting serum was used. The number of patients with type 2 diabetes and patients with liver steatosis was rather small and, therefore, current findings must be confirmed in larger study groups. 

## 4. Materials and Methods

### 4.1. Study Cohort

This study was scheduled at the Department of Internal Medicine I (University Hospital of Regensburg) from October 2014 to September 2019 [25,35]. Patients with chronic HCV infection, who were suitable for DAA therapy in accordance with recent guidelines [51], were enrolled in the study. The HCV treatment-naive patients signed an informed consent form and DAA therapy lasted for 12 weeks. The patients were older than 18 years and were not co-infected with the hepatitis B virus or human immunodeficiency virus.

The patients were treated with sofosbuvir/daclatasvir (74), sofosbuvir/ledipasvir (47), dasabuvir/ombitasvir/paritaprevir/ritonavir (30), sofosbuvir/ribavirin (10), sofosbuvir/ledipasvir/ribavirin (7), sofosbuvir/velpatasvir (6), or sofosbuvir/daclatasvir/ribavirin (4), following international treatment recommendations [51]. Laboratory values were provided by the Institute of Clinical Chemistry and Laboratory Medicine (University Hospital of Regensburg). Statins were paused during treatment. Liver cirrhosis was diagnosed by ultrasound, and here, liver surface, size, and parenchyma were analyzed [52]. Cut-off values for the FIB-4 score were the following: advanced fibrosis > 3.25, no fibrosis < 1.30 (for patients younger than 65 years), and no fibrosis < 2.00 (for patients older than 65 years) [53]. Patients’ characteristics are summarized in Table 1 of a recent, freely available publication [35]. 

### 4.2. Lipid Extraction and Analysis of SM Species

The internal standard SM 30:1;O2 (Avanti Polar Lipids, Alabaster, AL, USA) was added to the serum (0.39 nMol/sample). A volume of 10 µL serum was used for lipid extraction, which was performed in accordance with the protocol described by Bligh and Dyer [54], using a total chloroform volume of 2 mL. A volume of 1 mL of the chloroform phase was pipetted into sample vials by a pipetting robot (Tecan Genesis RSP 150, Männedorf, Switzerland) and vacuum-dried. The residues were solubilized in isopropanol/methanol (Merck, Darmstadt, Germany)/chloroform (Roth, Karlsruhe, Germany) with 7.5 mM ammonium formate. 

Lipids were quantified by direct flow injection analysis (FIA) using a hybrid quadrupole-Orbitrap mass spectrometer QExactive (Thermo Fisher Scientific, Bremen, Germany). The measurements were performed in negative ion mode in the *m*/*z* range 520–960 [55]. Quantification of ceramides in the serum of these patients has been described before [35].

### 4.3. Statistical Analysis

Data are shown as boxplots, which display the minimum value, the maximum value, the median, and the first and third quartiles. Circles/asterisks below or above the boxes mark outliers. Data are also shown as bar charts (mean concentration ± standard deviation). Data are reported as median values and the minimum and maximum values. The non-parametric Kruskal–Wallis test and Spearman’s correlation were used (SPSS Statistics 26.0 program). The Student’s *t*-test was used for paired data (MS Excel). Data were corrected for multiple comparisons. A value of *p* < 0.05 was considered significant.

## 5. Conclusions

The present study showed that the serum SM profile differs between sexes and is also affected by HCV infection and HCV genotype. Various SM species were low in liver cirrhosis in female and in male patients before and after DAA therapy. Strong correlations of SM species with the MELD score in males but not females with liver cirrhosis indicate a close association between SM metabolism and liver (dys)function in male patients. Dietary SM is beneficial in different diseases [33], and identification of species associated with chronic liver diseases could open up new therapeutic possibilities.

## Figures and Tables

**Table 1:** Spearman correlation coefficients for the associations of sphingomyelin (SM) species with model of end-stage liver disease (MELD) score, bilirubin, and international normalized ratio (INR); - not significant, * *p* < 0.05, ** *p* < 0.01, *** *p* < 0.001.

SM Species nmol/mL	MELD Score	Bilirubin mg/dL	INR
32:1;O2	-	-	-
33:1;O2	-	-	-
34:1;O2	-	-	-
34:2;O2	−0.629 *	-	-
35:1;O2	-	-	-
36:1;O2	−0.663 **	-	−0.636 *
36:2;O2	−0.724 **	-	−0.685 **
37:1;O2	−0.653 *	-	−0.750 ***
38:1;O2	−0.733 ***	−0.614 *	−0.749 ***
38:2;O2	−0.693 **	-	−0.642 *
39:1;O2	−0.612 *	-	−0.667 **
40:1;O2	−0.648 *	-	−0.666 **
40:2;O2	−0.651 *	-	−0.631 *
41:1;O2	−0.664 **	-	−0.672 **
41:2;O2	-	-	-
42:1;O2	−0.610 *	-	−0.612 *
42:2;O2	-	-	-
43:2;O2	−0.604 *	-	−0.612 *

**Table 2:** Levels of sphingomyelin (SM) species (median, minimum, and maximum values) of females with and without liver cirrhosis at the end of therapy. * *p* < 0.05, ** *p* < 0.01, *** *p* < 0.001.

SM nmol/mL	Median	Minimum	Maximum	Median	Minimum	Maximum	*p*-Value
	No Cirrhosis			Cirrhosis			
32:1;O2	11.13	6.33	21.07	9.86	5.89	16.02	
33:1;O2	6.75	3.83	13.16	5.83	2.84	9.23	
34:1;O2	122.02	91.74	200.48	116.07	74.81	173.25	
34:2;O2	16.27	8.35	28.16	13.02	6.65	19.27	*
35:1;O2	3.35	2.12	6.28	2.19	1.23	3.98	**
36:1;O2	23.25	12.53	42.51	14.97	8.99	28.35	***
36:2;O2	10.57	3.51	18.24	5.72	3.13	12.03	***
37:1;O2	1.72	0.77	3.39	0.99	0.10	2.09	**
38:1;O2	12.61	2.64	27.66	5.99	1.12	13.40	***
38:2;O2	6.39	2.00	10.84	3.85	1.88	7.15	***
39:1;O2	6.20	2.89	13.74	4.08	0.70	6.79	**
40:1;O2	30.27	15.33	54.87	21.02	7.86	27.51	***
40:2;O2	25.43	12.05	41.00	17.10	9.34	25.54	***
41:1;O2	13.39	7.49	29.82	10.21	1.87	13.31	***
41:2;O2	12.40	7.10	23.18	8.41	3.82	13.92	***
42:1;O2	18.06	9.44	37.61	12.34	5.73	17.58	***
42:2;O2	64.15	39.12	108.34	49.83	35.85	97.20	*
43:2;O2	3.25	1.51	7.03	2.15	1.13	4.01	*

**Table 3:** Levels of sphingomyelin (SM) species (median, minimum, and maximum values) of male patients with and without liver cirrhosis at the end of therapy. ** *p* < 0.01, *** *p* < 0.001.

SM nmol/mL	Median	Minimum	Maximum	Median	Minimum	Maximum	*p*-Value
	No Cirrhosis			Cirrhosis			
32:1;O2	9.57	5.43	19.18	9.02	5.43	16.87	
33:1;O2	5.61	3.44	11.84	5.60	2.26	8.69	
34:1;O2	120.40	75.18	178.35	112.46	75.12	174.24	
34:2;O2	13.74	8.36	22.51	10.92	4.36	19.75	**
35:1;O2	2.76	1.38	5.73	2.39	1.04	4.32	
36:1;O2	21.32	10.61	37.17	15.09	6.78	26.46	**
36:2;O2	8.72	3.88	14.85	5.19	2.37	9.79	***
37:1;O2	1.32	0.35	3.13	0.90	0.24	2.15	
38:1;O2	10.40	3.05	23.22	5.34	1.39	15.55	***
38:2;O2	5.39	2.30	9.57	3.37	1.10	5.88	***
39:1;O2	4.93	2.74	10.88	3.44	1.21	8.89	**
40:1;O2	29.01	16.88	45.71	18.08	7.27	32.44	***
40:2;O2	21.41	11.55	36.39	14.41	8.92	26.34	***
41:1;O2	12.48	7.59	20.69	8.63	2.99	17.91	***
41:2;O2	10.38	5.54	19.64	7.60	5.07	14.46	**
42:1;O2	18.42	10.78	29.76	11.77	5.74	20.73	***
42:2;O2	65.88	39.16	122.82	50.87	35.44	92.66	
43:2;O2	2.90	1.36	6.10	2.06	1.19	4.65	

## Data Availability

Original data can be obtained from the corresponding author.

## Supplementary Materials

The following supporting information can be downloaded at: https://www.mdpi.com/article/10.3390/ijms24098402/s1.
