# Supplementary material for: Gender-Specific Differences in Serum Sphingomyelin Species in Patients with Hepatitis C Virus Infection—Sphingomyelin Species Are Related to the Model of End-Stage Liver Disease (MELD) Score in Male Patients
Source: Int J Mol Sci. 2023 May 7;24(9):8402. doi: 10.3390/ijms24098402 (PMC10179471; doi:10.3390/ijms24098402)
Supplement: Supplementary file 1 [file ijms-24-08402-s001.zip › ijms-2365736-supplementary.pdf]

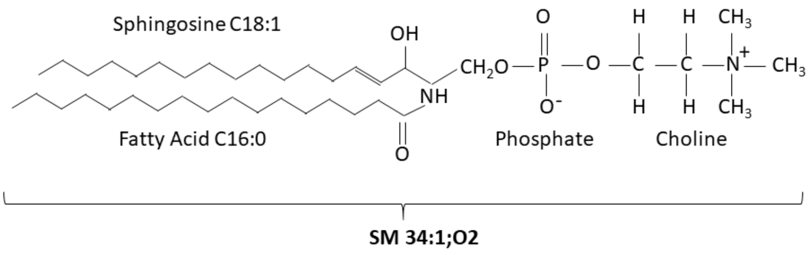

Figure S1. General structure of sphingomyelin.

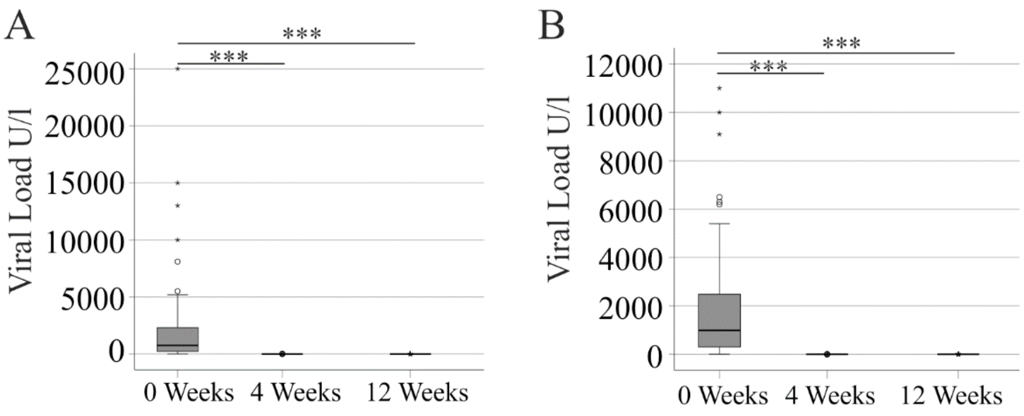

Figure S2. Viral load during the study. Viral titer did not differ between females and males (p = 0.535, 0.412 and 0.877 at 0, 4 and 12 weeks, respectively. (**A**) Viral load in females during the study; (**B**) Viral load in males during the study. *** p < 0.001

Table S1. SM species in serum of female and male patients with and without type 2 diabetes. * $p < 0.05$. Significantly different lipid species levels are in bold.

	Females, no diabetes			Females, diabetes			Males, no diabetes			Males, diabetes		
**SM nmol/mL**	**Median**	**Min**	**Max**	**Median**	**Min**	**Max**	**Median**	**Min**	**Max**	**Median**	**Min**	**Max**
32:1	9.97	5.62	20.09	9.16	5.89	15.16	8.74	2.44	18.69	9.87	4.48	14.45
33:1	5.80	2.86	11.05	6.33	3.21	9.04	4.91	1.29	10.69	5.79	3.26	8.92
34:1	110.46	76.48	175.70	109.45	93.82	133.11	109.24	50.81	168.69	112.60	75.07	159.92
34:2	13.45	4.53	23.92	12.38	11.01	16.50	12.73	4.10	18.59	11.30	5.57	16.65
35:1	2.92	1.16	5.86	2.38	1.48	3.59	2.57	0.36	5.63	2.64	1.60	3.53
36:1	21.15	7.59	34.04	13.50	11.37	18.50	18.71	5.18	33.42	15.42	7.38	21.26
36:2	9.43	2.64	15.45	6.13	4.99	8.61	**7.91***	**1.83**	**12.75**	**5.48***	**3.33**	**8.34**
37:1	1.53	0.20	3.06	0.88	0.39	1.38	1.07	0.00	2.97	1.07	0.11	1.56
38:1	8.85	0.99	20.56	4.90	2.65	6.37	7.32	0.84	19.23	5.88	1.25	9.97
38:2	5.54	1.56	9.78	3.86	3.05	4.34	**4.42***	**1.30**	**7.74**	**3.37***	**1.56**	**5.01**
39:1	4.78	0.91	11.31	3.92	1.99	5.16	3.89	1.19	9.31	3.84	1.93	5.89
40:1	24.65	7.73	42.61	19.46	15.72	20.08	23.67	7.22	44.66	20.58	11.27	29.88
40:2	21.37	8.57	33.04	14.94	13.32	20.34	18.05	7.98	32.57	15.02	8.13	27.45
41:1	10.37	1.95	23.65	8.56	6.13	11.15	9.46	2.93	19.76	9.68	5.45	14.66
41:2	10.25	3.63	19.54	7.92	5.73	10.68	8.54	2.82	18.73	8.18	4.00	14.38
42:1	14.38	5.80	28.04	10.65	9.56	13.90	15.17	5.21	30.73	13.41	8.11	23.77
42:2	57.83	33.51	84.58	51.53	40.77	58.29	56.32	23.97	109.30	53.88	33.96	100.65
43:2	2.78	1.09	6.47	2.39	1.50	3.00	2.60	0.59	6.34	2.66	1.21	5.41

Table S2. Sphingomyelin species in serum of 33 females without liver fibrosis, 24 females with intermediate and 17 females with high fibrosis scores. * $p < 0.05$, ** $p < 0.01$ and *** $p < 0.001$ for the comparison of No Fibrosis and Fibrosis. § $p < 0.05$, §§ $p < 0.01$ and §§§ $p < 0.001$ for the comparison of Intermediate scores and Fibrosis.

	**No Fibrosis**			**Intermediate**			**Fibrosis**			***P*-value**
**SM species nmol/ml**	**Median**	**Min**	**Max**	**Median**	**Min**	**Max**	**Median**	**Min**	**Max**	
32:1	10.02	6.27	16.59	13.05	6.20	20.09	8.84	5.62	15.16	
33:1	5.74	3.96	9.22	7.00	3.24	11.05	5.43	2.86	9.04	
34:1	116.27	90.06	139.47	126.51	84.54	175.70	101.89	76.48	133.11	§
34:2	15.23	9.82	21.45	15.79	9.34	23.92	11.71	4.53	16.50	*§
35:1	3.06	1.82	4.70	3.26	1.49	5.86	2.12	1.16	3.59	§§
36:1	22.20	15.82	33.16	22.49	9.71	34.04	13.50	7.59	20.03	***§§§
36:2	10.07	5.52	15.45	10.17	3.65	14.13	6.13	2.64	8.61	***§§§
37:1	1.56	0.44	2.87	1.74	0.23	3.06	0.52	0.20	1.97	*§§
38:1	8.96	4.48	14.38	11.36	1.84	20.56	4.24	0.99	9.66	***§§§
38:2	5.73	3.05	8.39	5.88	2.04	9.78	3.86	1.56	4.66	***§§§
39:1	4.90	2.39	7.95	6.25	1.95	11.31	2.88	0.91	5.87	*§§
40:1	25.22	16.68	33.36	27.34	12.61	42.61	17.78	7.73	23.45	§§
40:2	22.59	13.35	28.72	23.89	10.20	33.04	14.87	8.57	20.57	***§§§
41:1	11.15	6.13	15.12	12.37	5.64	23.65	7.43	1.95	10.37	***§§§
41:2	10.78	5.82	15.43	11.90	6.22	19.54	7.41	3.63	10.68	***§§§
42:1	14.89	9.86	23.44	16.16	8.49	28.04	10.46	5.80	13.90	***§§§
42:2	58.19	44.26	74.61	61.98	37.93	84.58	43.99	33.51	70.38	***§§§
43:2	2.83	1.44	5.05	2.95	1.55	6.47	2.22	1.09	3.33	§§§

Table S3. Spearman correlation coefficients for the associations of sphingomyelin species with the MELD score and routine laboratory parameters in female HCV patients without liver cirrhosis before direct-acting antiviral (DAA) therapy. Significant correlations are in bold (alanine aminotransferase (ALT), aspartate aminotransferase (AST), C-reactive protein (CRP), international normalized ratio (INR), model of end-stage liver disease (MELD) score. * $p < 0.05$.

**SM species nmol/ml**	MELD score	ALT U/L	AST U/L	Bilirubin mg/dL	Albumin g/L	INR	CRP mg/L	Leukocytes n/L	Platelets n/nL	Creatinine mg/dL
32:1	−0.304	−0.197	−0.143	0.111	0.026	−0.320	0.087	−0.105	−0.108	0.133
33:1	−0.186	−0.226	−0.174	0.219	0.047	−0.228	0.220	−0.125	−0.170	0.165
34:1	−0.268	−0.197	−0.146	0.150	0.091	−0.298	0.280	−0.037	−0.132	0.221
34:2	**−0.417***	−0.274	−0.246	0.079	0.212	**−0.440***	0.210	−0.047	0.073	0.071
35:1	−0.158	−0.148	−0.228	0.143	0.054	−0.182	0.234	0.004	−0.122	0.162
36:1	−0.061	−0.185	−0.209	0.084	0.094	−0.104	0.311	0.169	0.068	0.227
36:2	−0.276	−0.239	−0.275	0.129	0.207	−0.302	0.235	0.126	0.114	0.163
37:1	−0.075	−0.148	−0.204	0.160	0.074	−0.119	0.241	0.064	−0.084	0.249
38:1	−0.248	−0.272	−0.249	0.134	0.094	−0.280	0.111	−0.064	−0.028	0.143
38:2	−0.282	−0.241	−0.242	0.164	0.204	−0.310	0.143	0.088	0.174	0.173
39:1	−0.216	−0.224	−0.236	0.136	0.080	−0.254	0.120	−0.161	−0.080	0.139
40:1	−0.323	−0.246	−0.291	0.096	0.079	−0.338	0.187	−0.133	−0.077	0.111
40:2	−0.372	−0.291	−0.281	0.154	0.216	−0.387	0.173	−0.125	0.073	0.113
41:1	−0.192	−0.133	−0.185	0.170	0.022	−0.261	0.265	−0.200	−0.174	0.158
41:2	−0.249	−0.232	−0.234	0.180	0.173	−0.298	0.174	−0.133	0.011	0.133
42:1	−0.221	−0.115	−0.184	0.064	0.026	−0.217	0.161	−0.285	−0.139	0.161
42:2	−0.142	−0.122	−0.079	0.193	0.171	−0.164	0.285	−0.088	−0.033	0.242
43:2	0.018	0.002	−0.005	0.253	0.078	−0.023	0.232	−0.108	−0.283	0.209

Table S4. Spearman correlation coefficients for the associations of sphingomyelin species with the MELD score and routine laboratory parameters in female HCV patients with liver cirrhosis before direct-acting antiviral (DAA) therapy. Significance level was set to $p < 0.05$. Significant correlations are in bold (alanine aminotransferase (ALT), aspartate aminotransferase (AST), C-reactive protein (CRP), international normalized ratio (INR), model of end-stage liver disease (MELD) score. * $p < 0.05$, ** $p < 0.01$.

SM species nmol/ml	MELD score	ALT U/L	AST U/L	Bilirubin mg/dL	Albumin g/L	INR	CRP mg/L	Leukocytes n/L	Platelets n/nL	Creatinine mg/dL
32:1;O2	0.256	−0.571	−0.203	−0.065	0.365	−0.113	0.037	0.285	−0.094	−0.043
33:1;O2	−0.049	−0.500	−0.124	−0.361	.503	−0.271	0.000	0.394	0.131	0.112
34:1;O2	−0.314	−0.406	−0.091	−0.540	0.475	−0.405	0.328	0.418	0.212	0.138
34:2;O2	−0.149	−0.538	−0.479	−0.168	0.537	−0.186	0.400	−0.018	−0.225	−0.211
35:1;O2	−0.255	−0.476	−0.068	−0.597	0.678	−0.336	0.184	0.218	0.107	−0.022
36:1;O2	−0.267	−0.515	−0.106	−0.656	0.642	−0.360	0.238	0.179	0.153	−0.186
36:2;O2	−0.414	−0.459	−0.200	−0.580	**0.815****	−0.370	0.334	0.029	0.047	−0.276
37:1;O2	−0.136	−0.571	−0.162	−0.577	0.701	−0.262	0.313	0.074	0.007	−0.177
38:1;O2	−0.179	−0.497	−0.079	−0.533	0.695	−0.389	0.201	0.365	0.106	−0.108
38:2;O2	−0.311	−0.432	−0.224	−0.418	**0.768***	−0.280	0.437	−0.056	−0.116	−0.406
39:1;O2	−0.133	−0.450	−0.026	−0.455	0.614	−0.405	0.106	0.374	0.213	−0.177
40:1;O2	−0.249	−0.488	−0.068	−0.565	0.661	−0.508	0.357	0.444	0.268	−0.043
40:2;O2	−0.210	−0.506	−0.112	−0.493	0.687	−0.450	0.301	0.344	0.159	−0.173
41:1;O2	−0.301	−0.444	−0.021	−0.603	0.677	−0.547	0.303	0.468	0.334	−0.078
41:2;O2	−0.368	−0.526	−0.147	−0.580	0.681	−0.576	0.226	0.485	0.334	0.009
42:1;O2	−0.377	−0.232	0.103	−0.562	0.509	−0.507	0.282	0.482	0.337	−0.121
42:2;O2	−0.414	−0.265	−0.185	−0.302	0.188	−0.495	0.325	0.432	0.336	0.160
43:2;O2	−0.359	−0.268	0.135	−0.631	0.474	−0.470	0.164	0.497	0.343	0.151

Table S5. Correlation of SM species with HDL and LDL in female and male patients with cirrhosis before DAA therapy. * $p < 0.05$, ** $p < 0.01$, *** $p < 0.001$.

**SM species nmol/ml**	HDL mg/dl	LDL mg/dl	HDL mg/dl	LDL mg/dl
	**Females**		**Males**	
32:1	0.322	0.375	0.529	**0.724****
33:1	0.286	0.582	0.544	**0.748****
34:1	0.209	**0.932*****	0.669	0.702
34:2	0.606	0.364	0.449	**0.827*****
35:1	0.488	0.629	0.585	**0.851*****
36:1	0.601	0.650	0.634	**0.841*****
36:2	**0.754***	0.521	0.496	**0.852*****
37:1	0.663	0.625	0.505	**0.852*****
38:1	0.493	**0.736***	0.496	**0.903*****
38:2	**0.790***	0.479	0.567	**0.818*****
39:1	0.509	0.568	0.474	**0.880*****
40:1	0.441	**0.868****	0.563	**0.909*****
40:2	0.620*	0.718	0.480	**0.924*****
41:1	0.454	0.829	0.494	**0.919*****
41:2	0.567	0.714	0.520	**0.894*****
42:1	0.307	**0.839****	0.563	**0.841*****
42:2	0.093	0.586	0.660	**0.764*****
43:2	0.297	0.764	0.500	**0.853*****

Table S6. Sphingomyelin species in serum of 43 males without fibrosis, 28 males with intermediate and 33 males with high fibrosis scores. ** $p < 0.01$ and *** $p < 0.001$ for the comparison of No Fibrosis and Fibrosis. § $p < 0.05$ for the comparison of Intermediate scores and Fibrosis.

	**No Fibrosis**			**Intermediate**			**Fibrosis**			***p*-value**
**SM species nmol/ml**	**Median**	**Min**	**Max**	**Median**	**Min**	**Max**	**Median**	**Min**	**Max**	
32:1	8.89	2.44	16.33	9.90	4.00	16.07	8.82	4.35	18.69	
33:1	4.91	2.08	9.81	5.67	2.33	9.15	5.02	1.29	10.69	
34:1	110.98	50.81	168.69	109.73	55.85	154.65	108.89	57.59	159.92	
34:2	12.85	5.83	18.59	12.23	6.93	18.17	11.16	4.10	16.65	
35:1	2.61	1.02	5.28	2.90	1.18	4.96	2.25	0.36	5.63	
36:1	19.69	5.18	29.10	19.76	8.96	26.46	14.78	5.43	33.42	**
36:2	8.15	3.05	12.75	7.67	4.14	11.73	4.89	1.83	12.07	***§
37:1	1.07	0.04	2.82	1.36	0.24	2.47	0.76	0.00	2.97	
38:1	7.48	0.84	18.26	8.31	2.78	15.71	5.23	1.25	19.23	***§
38:2	4.59	2.22	7.74	4.63	2.33	7.18	3.38	1.30	6.83	**
39:1	4.25	1.44	9.31	4.17	1.75	8.13	3.48	1.19	9.13	
40:1	25.22	13.13	44.66	23.75	12.36	33.27	19.52	7.22	29.88	***
40:2	18.26	10.50	32.57	19.39	10.46	24.86	14.67	7.98	28.51	**
41:1	9.56	3.98	19.76	10.48	4.18	13.84	8.35	2.93	14.66	
41:2	8.54	3.38	18.73	9.46	4.52	14.21	7.28	2.82	14.38	
42:1	16.15	9.63	30.73	15.28	6.53	21.38	12.16	5.21	23.77	**
42:2	58.80	26.20	109.30	52.39	23.97	88.49	53.48	29.24	100.65	
43:2	2.57	0.59	6.34	3.00	0.81	5.34	2.01	0.67	5.41	

Table S7. Spearman correlation coefficients for the associations of sphingomyelin species with MELD score and routine laboratory parameters in male HCV patients with liver cirrhosis before direct-acting antiviral (DAA) therapy. Significance level was set to $p < 0.05$. Significant correlations are in bold (alanine amino transferase (ALT), aspartate aminotransferase (AST), C-reactive protein (CRP), international normalized ratio (INR), model of end-stage liver disease (MELD). * $p < 0.05$, ** $p < 0.01$, *** $p < 0.001$.

SM species nmol/ml	MELD score	ALT U/L	AST U/L	Bilirubin mg/dL	Albumin g/L	INR	CRP mg/L	Leukocytes n/L	Platelets n/nL	Creatinine mg/dL
32:1;O2	−0.340	0.167	0.204	−0.197	−0.003	−0.225	0.259	0.342	0.268	0.017
33:1;O2	−0.353	0.298	0.293	−0.217	0.082	−0.329	0.180	0.404	0.301	0.013
34:1;O2	−0.249	0.010	−0.020	−0.201	0.019	−0.236	−0.114	0.351	0.240	−0.095
34:2;O2	**−0.629***	0.024	−0.123	−0.484	0.246	−0.503	0.162	0.262	0.320	−0.128
35:1;O2	−0.507	.430	0.234	−0.358	0.271	−0.499	0.084	0.440	0.381	−0.101
36:1;O2	**−0.663****	0.388	0.087	−0.489	0.470	**−0.636***	−0.112	0.427	0.480	−0.283
36:2;O2	**−0.724****	0.241	−0.071	−0.560	0.471	**−0.685****	0.048	0.331	0.394	−0.216
37:1;O2	**−0.653***	0.589	0.214	−0.509	0.495	**−0.750*****	0.069	0.477	0.451	−0.042
38:1;O2	**−0.733*****	0.455	0.068	**−0.614***	0.507	**−0.749*****	−0.019	0.486	0.530	−0.126
38:2;O2	**−0.693****	0.309	0.043	−0.565	0.424	**−0.642***	0.100	0.368	0.385	−0.176
39:1;O2	**−0.612***	0.501	0.185	−0.476	0.379	**−0.667****	0.172	0.455	0.434	0.036
40:1;O2	**−0.648***	0.409	0.052	−0.496	0.418	**−0.666****	−0.012	0.469	0.518	−0.139
40:2;O2	**−0.651***	0.288	0.023	−0.496	0.376	**−0.631***	0.154	0.452	0.477	−0.072
41:1;O2	**−0.664****	0.465	0.112	−0.544	0.435	**−0.672****	0.002	0.489	0.521	−0.055
41:2;O2	−0.588	0.359	0.151	−0.412	0.279	−0.573	0.160	0.486	0.391	−0.021
42:1;O2	**−0.610***	0.428	0.093	−0.526	0.348	**−0.612***	−0.102	0.524	0.513	−0.093
42:2;O2	−0.439	0.198	0.129	−0.278	0.109	−0.399	0.073	0.432	0.361	−0.169
43:2;O2	**−0.604***	0.436	0.205	−0.461	0.252	**−0.612***	0.215	0.484	0.452	0.039

Table S8. Spearman correlation coefficients for the associations of sphingomyelin species with the MELD score and routine laboratory parameters in male HCV patients without liver cirrhosis before direct-acting antiviral (DAA) therapy. Significant correlations were not identified (alanine aminotransferase (ALT), aspartate aminotransferase (AST), C-reactive protein (CRP), international normalized ratio (INR), model of end-stage liver disease (MELD).

SM species nmol/ml	MELD score	ALT U/L	AST U/L	Bilirubin mg/dL	Albumin g/L	INR	CRP mg/L	Leukocytes n/L	Platelets n/nL	Creatinine mg/dL
32:1	0.062	0.061	0.081	0.001	−0.092	0.005	0.131	−0.072	0.047	0.141
33:1	−0.002	−0.050	−0.024	−0.031	0.074	−0.069	0.111	−0.156	0.076	0.117
34:1	−0.105	−0.062	−0.064	−0.014	0.078	−0.065	0.154	−0.089	0.113	0.019
34:2	−0.062	−0.084	−0.074	0.017	0.099	−0.130	0.186	−0.184	−0.018	0.176
35:1	−0.122	−0.163	−0.150	−0.113	0.221	−0.162	0.071	−0.094	0.178	0.082
36:1	−0.226	−0.153	−0.190	−0.151	0.241	−0.244	0.129	−0.010	0.238	0.030
36:2	−0.240	−0.224	−0.261	−0.158	0.302	−0.289	0.043	−0.088	0.187	0.091
37:1	−0.051	−0.138	−0.105	−0.100	0.207	−0.112	0.026	−0.124	0.146	0.083
38:1	−0.034	−0.065	−0.139	−0.089	0.171	−0.150	0.130	−0.109	0.214	0.115
38:2	−0.111	−0.213	−0.244	−0.137	0.225	−0.197	0.078	−0.122	0.128	0.098
39:1	0.072	−0.048	−0.038	−0.035	0.038	−0.047	0.055	−0.203	0.097	0.125
40:1	−0.123	−0.051	−0.127	−0.048	0.142	−0.212	0.167	−0.146	0.251	0.048
40:2	−0.115	−0.127	−0.165	−0.104	0.144	−0.200	0.140	−0.159	0.164	0.096
41:1	−0.063	−0.038	−0.048	−0.099	0.077	−0.127	0.149	−0.195	0.164	0.106
41:2	−0.072	−0.126	−0.134	−0.106	0.098	−0.135	0.099	−0.194	0.106	0.104
42:1	−0.120	0.035	−0.051	−0.008	0.052	−0.211	0.232	−0.084	0.278	0.032
42:2	−0.284	−0.126	−0.176	−0.135	0.043	−0.213	0.268	0.052	0.262	−0.083
43:2	−0.164	−0.084	−0.044	−0.217	0.093	−0.152	0.093	−0.106	0.165	0.030

Table S9. Sphingomyelin species in relation to total sphingomyelin levels in serum of females with and without liver cirrhosis at therapy end. * $p < 0.05$, ** $p > 0.01$ and *** $p < 0.001$. ↑ higher in cirrhosis, ↓ lower in cirrhosis.

% SM species	Median	Minimum	Maximum	Median	Minimum	Maximum	*p*-value
	No Cirrhosis			Cirrhosis			
% 32:1	3.08	2.01	4.02	3.59	2.05	4.67	
% 33:1	1.71	1.22	2.34	1.97	1.19	2.59	
% 34:1	31.25	28.47	41.56	36.65	33.80	44.24	*** ↑
% 34:2	4.17	3.11	5.97	4.09	3.14	6.20	
% 35:1	0.87	0.63	1.20	0.76	0.53	1.09	
% 36:1	5.92	4.31	7.93	5.15	3.69	6.52	** ↓
% 36:2	2.70	1.32	3.71	2.03	1.30	2.50	*** ↓
% 37:1	0.45	0.20	0.70	0.26	0.05	0.64	* ↓
% 38:1	3.20	0.99	4.39	2.03	0.59	3.61	*** ↓
% 38:2	1.67	0.75	2.15	1.21	0.78	1.72	*** ↓
% 39:1	1.63	1.04	2.27	1.39	0.37	2.08	
% 40:1	7.50	5.50	10.64	6.60	4.15	7.86	** ↓
% 40:2	6.41	4.53	7.64	5.62	4.08	6.43	*** ↓
% 41:1	3.51	2.48	4.51	3.04	0.99	3.75	*↓
% 41:2	3.18	2.45	3.94	2.80	2.02	3.41	* ↓
% 42:1	4.48	3.21	7.28	3.72	3.03	5.55	* ↓
% 42:2	16.42	11.87	19.98	17.19	12.54	22.83	
% 43:2	0.83	0.43	1.46	0.72	0.51	1.04	

Table S10. Sphingomyelin species in relation to total sphingomyelin levels in serum of males with and without liver cirrhosis at therapy end. * $p < 0.05$, ** $p > 0.01$ and *** $p < 0.001$ ↑ higher in cirrhosis, ↓ lower in cirrhosis.

%SM species	Median	Minimum	Maximum	Median	Minimum	Maximum	*p*-value
	No Cirrhosis			Cirrhosis			
% 32:1	2.70	1.70	4.48	3.29	2.24	4.35	* ↑
% 33:1	1.59	0.98	2.50	1.94	0.97	2.79	** ↑
% 34:1	32.47	28.43	38.72	38.34	29.79	43.57	*** ↑
% 34:2	3.71	3.00	4.75	3.65	1.71	4.69	
% 35:1	0.80	0.45	1.26	0.82	0.45	1.07	
% 36:1	5.81	4.35	7.85	5.31	3.57	6.46	* ↓
% 36:2	2.43	1.70	3.64	1.82	0.93	2.60	*** ↓
% 37:1	0.36	0.16	0.67	0.32	0.11	0.56	
% 38:1	2.82	1.36	4.57	1.91	0.73	3.86	*** ↓
% 38:2	1.45	0.92	2.02	1.14	0.43	1.73	*** ↓
% 39:1	1.34	0.79	2.31	1.15	0.64	2.20	
% 40:1	7.85	6.28	10.71	6.30	3.83	8.37	*** ↓
% 40:2	5.84	4.72	7.53	5.04	3.56	6.71	*** ↓
% 41:1	3.40	2.49	4.42	2.89	1.57	4.44	** ↓
% 41:2	2.82	2.14	3.60	2.55	2.09	3.59	
% 42:1	5.05	3.33	7.39	3.98	3.02	5.41	*** ↓
% 42:2	17.50	11.28	21.98	18.19	14.09	21.95	
% 43:2	0.77	0.47	1.20	0.76	0.51	1.15	
